# The expanded prostate cancer index composite short form (EPIC-26) for measuring health-related quality of life: content analysis of patients’ spontaneous comments written in survey margins

**DOI:** 10.1007/s11136-021-02940-z

**Published:** 2021-07-21

**Authors:** Anna-Maija Talvitie, Hanna Ojala, Teuvo Tammela, Ilkka Pietilä

**Affiliations:** 1 Faculty of Social Sciences, Tampere University, Tampere, Finland; 2 Faculty of Medicine and Health Technology, Tampere University; Tampere University Hospital, Tampere, Finland; 3 Faculty of Social Sciences, University of Helsinki, Helsinki, Finland

**Keywords:** The expanded prostate cancer index composite short form, Quality of life, Prostate cancer, Qualitative methods, Content analysis, Patient experience

## Abstract

**Introduction:**

This study investigates comments that prostate cancer patients spontaneously write in the margins of the Expanded Prostate Cancer Index Short Form (EPIC-26) questionnaire. We aim to show the possible barriers that patients face while answering the survey, and to consider how these barriers may affect the response data generated. We investigate the kind of information patients’ comments on EPIC-26 contain, and patients’ motivations to provide this information. We also study why some EPIC domains spark more comments than others.

**Method:**

We analyzed 28 pages of transcribed comments and four pages of supplementary letters from our survey participants (*n* = 496). Using inductive content analysis, we generated 10 categories describing the content of participants’ comments, and four themes demonstrating their motives for commenting. The comments regarding each EPIC domain were quantified to discover any differences between domains.

**Results:**

The sexual domain of EPIC-26 provoked over half of all comments. Patients without recent sexual activity or desire had difficulties answering sexual function questions 8–10. The lack of instructions on whether to take erectile aid use into account when answering erectile function questions led to a diversity of answering strategies. Patients with urinary catheters could not find suitable answer options for questions 1–4. All domains sparked comments containing additional information about experienced symptoms.

**Conclusion:**

Patients are mainly willing to report their symptoms, but a lack of suitable answer options causes missing data and differing answering strategies in the sexual and urinary domains of EPIC-26, weakening the quality of the response data received.

**Supplementary Information:**

The online version contains supplementary material available at 10.1007/s11136-021-02940-z.

## Introduction

Measurement instruments are necessary tools for research and clinical practice [1]. Choosing a quantitative instrument to assess patient-reported outcomes, however, can be challenging, since all research instruments have limitations. Measuring quality of life (QoL) is particularly challenging. QoL is subjective, multidimensional, and complex, and even the best instruments can capture only a slice of its entirety [2, 3]. Health-related QoL (HRQoL) includes physical, psychological, and social dimensions that impact one’s health [4]. Assessment of HRQoL is particularly important in healthcare, since many diseases and treatments can affect patients’ perceived health and well-being. This is especially important regarding diseases where treatment decisions may be affected by expected QoL outcomes [3]. Therefore, it is essential that QoL instruments are valid, reliable, and psychometrically sound [4].

Several validated and extensively used QoL instruments have proved suitable for HRQoL evaluation with prostate cancer patients. The most frequently used HRQoL instruments are the 50-item Expanded Prostate Cancer Index Composite (EPIC-50) [5], its short form (EPIC-26) [6, 7], and the 16-item EPIC for Clinical Practice (EPIC-CP) [8] for routine clinical use. These instruments include five symptom domains—urinary incontinence, urinary irritation, bowel, sexual, and hormonal—that relate to common prostate cancer treatment side effects and related bother. EPIC-26, which was also used in this study, includes 26 items: three items for urinary function and six for urinary bother, six items for bowel bother, five items for sexual function and one for sexual bother, and five items for hormonal bother. No validity or reliability issues have been found within this instrument [7]. Although EPIC-50 was developed in cooperation with patient experts [5], documentation of users’ experience with these EPIC-50-based instruments is scarce.

Korzeniowski et al. [9] interviewed clinicians about their experiences of using EPIC-26 in practice, perceptions of the instrument’s value, operational issues, and impressions of patient acceptability and value. These clinicians’ patients’ comments on ease of use and sources of confusion were also recorded. The authors found that patients were mostly willing to report their disease-specific symptoms to their healthcare team, since they felt this would affect their cancer care positively. The study revealed that a few patients with pretreatment sexual limitations faced barriers when answering questions related to sexual functioning, as a “not applicable” category was missing.

Because, to our knowledge, Korzeniowski et. al. are the only researchers to address user experience of EPIC-26 so far, we checked what there is to learn about other EPIC-50-based measures. Brundage et al. [10] evaluated the implementation of EPIC-CP in clinical practice for men with early-stage prostate cancer by exploring the usability and acceptability of the questionnaire through a patient “exit survey.” They also interviewed clinicians about EPIC-CP use. This multimethod study confirmed that the “majority of patients rated use of EPIC-CP favorably and also clinicians’ attitudes were supportive.” However, information about pragmatic usability was limited to patients’ survey answers regarding touchscreen or software use, font size, printing the report, images, place of answering the survey, and help received to answer the survey. A need for further information about why patients skip questions was recognized, although item completion rates were generally high (91–100%). In relation to our analysis, it is notable that the lowest completion rates in their study were for questions concerning sexual health.

We chose to use EPIC-26 in our research project evaluating long-term QoL changes among prostate cancer patients because it was already highly used in prostate cancer care in Finland. To our surprise, 22% of survey participants wrote comments in the margins of the EPIC questionnaire. The large number of remarks beside EPIC questions led us to consider whether men might have difficulties finding suitable answer options, or whether the questionnaire neglects matters that patients consider significant. Unlike studies concerning physicians’ views of EPIC, no previously published research uses qualitative methods to explore patients’ perceptions of EPIC instruments. Considering respondents’ feedback is crucial for the development of any instrument’s reliability and validity. Therefore, in this article, we aim to depict patients’ perspectives on the use of EPIC-26 (see Online Appendix).

## Aim

This study investigates the comments that prostate cancer patients write in the margins of the EPIC-26 questionnaire. Our goal is to use spontaneously generated qualitative data to show the possible barriers that patients face while answering EPIC-26, and to consider how these barriers may affect the response data generated from the instrument. Based on our findings, we also suggest some future directions to improve the quality of the instrument and minimize the number of skipped questions.

Our research questions are as follows:

1. What kind of information do patients’ comments on EPIC-26 contain, and what motivates patients to provide this information?
2. How do the numbers of comments differ between EPIC domains, and why do some domains spark more comments than others?

## Methods

### Study design

This study is part of an ongoing multimethod research project (2017–2023) that investigates patients’ QoL from cancer diagnosis to three years post diagnosis. The project combines a longitudinal survey and repeated interviews with newly diagnosed prostate cancer patients in one hospital district in Finland. The interview data were not used in this particular study. The patients were sent a questionnaire with a consent form and a study brochure by post. The monthly response rate in the first survey round varied between 47 and 74% (mean 62%). After the baseline survey, all study participants were sent the same questionnaire at six, 12, and 36 months post diagnosis (four survey rounds). Since recruitment closed at the end of 2020, and follow-up times range from zero to three years, the number of questionnaires completed by each patient varies.

### Patients and data

In total, 496 prostate cancer patients had been recruited for this study by August 6, 2020, when the data for these analyses were gathered. All 496 men had completed the first questionnaire, 352 had completed the second, and 216 the third. No patients had yet completed the final questionnaire (36 months post diagnosis). Our data consist of comments that survey participants wrote in the margins of our questionnaire. In total, 111 of our 496 participants (22%) made at least one EPIC-related comment at least once during the follow-up. Half of the patients commenting on EPIC questions wrote more than one comment on a single questionnaire, or commented several times during their participation. Mean age of these 111 men was 74 years (min = 50, max = 92). Majority of the men were married and retired. The patients’ education level varied (Table 1).

**Table 1:** Patient characteristics

Marital status	*n*	%
Married	78	70.3
Unmarried	9	8.1
Divorced	16	14.4
Widower	7	6.3
Missing	1	0.9
Education level		
No vocational education	15	13.5
Short vocational education	29	26.1
Vocational education	23	20.7
Polytechnic	24	21.6
University education	19	17.1
Missing	1	0.9
Labor force status		
Working full-time or part-time	16	14.4
Retired	92	82.9
Unemployed	0	0.0
Outside of the labor force for another reason	1	0.9
Missing	2	1.8

All the comments on the 1064 questionnaires were transcribed verbatim into a separate document (28 pages), and questions regarding disease-related symptoms and bother (EPIC) were then further analyzed. Two patients also wrote letters (four pages in total) to the researchers to supplement their comments and survey answers. These data were used similarly to the questionnaire comments during the analysis.

All study participants gave written informed consent. In addition, the two men who spontaneously wrote letters to the researchers gave us verbal consent to use the letters as part of our data. This verbal consent was recorded during their individual interviews that, however, were not used as research material in this study. All identifiable information such as treatment dates are changed in Table 2, which presents original data extracts, to ensure patients’ anonymity. The study received research permission from the university hospital and was approved by the ethics committee of the hospital district (5-10-2017).

**Table 2:** Patients’ EPIC-related comments and motives for commenting

Theme	Category (number of comments)	Sample extract (survey answer)	Referred EPIC domain	Referred EPIC question
Indicating difficulties in answering	No attempts at erection (36)	*"We don't have any sexual activity or need for such"* (no answer)	Sexual	11. Overall, how would you rate your ability to function sexually during the last 4 weeks?
		*"I have not wanted one"* (I never had an erection when I wanted one)	Sexual	10. How would you describe the FREQUENCY of your erections during the last 4 weeks?
		*"No experience from the past four weeks"* (fair)	Sexual	8b. How would you rate your ability to reach orgasm during the last 4 weeks?
	Use of medical devices (16)	*"Catheter"* (no answers)	Urinary	1–3. Urinary incontinence questions
		*"Cystofix"* (total control)	Urinary	2. Which of the following best describes your urinary control during the last 4 weeks?
		*"I had a colostomy after radiation therapy"* (no answers)	Bowel	6 a-e. Bowel bother questions
	Reluctance to answer (10)	*"What is dead is dead"* (no answers)	Sexual	All sexual function and bother questions
		*"NO!"* (no answers)	Sexual	8 a-b. How would you rate your ability to have an erection/reach orgasm during the last 4 weeks? 10. How would you describe the FREQUENCY of your erections during the last 4 weeks?
		*"NO THIS IS UNCOMFORTABLE FOR ME"* (no answers)	Sexual	All sexual function and bother questions
	Absence of a sexual relationship (6)	*My wife was buried the same week my possible cancer was found and afterwards confirmed with a biopsy. Sexual matters have been irrelevant in my life lately.* (Answered all sexual function question indicating poor sexual function and no bother)	Sexual	All sexual function and bother questions
		*"No sexual relationship"* (no answers)	Sexual	8 a-b. How would you rate your ability to have an erection/reach orgasm during the last 4 weeks? 10. How would you describe the FREQUENCY of your erections during the last 4 weeks?
		*" Also, estimates about sexual function during the last four weeks are rarely theoretical considering that I'm not in a relationship. Of course, I could answer that there is no problem, but the answer would hardly be very valid for this research."* (no answers)	Sexual	Excerpt from a letter explaining his sexual function
Explaining life situations	Old age (9)	*"Not very relevant anymore at this age"* (I never had an erection when I wanted one)	Sexual	10. How would you describe the FREQUENCY of your erections during the last 4 weeks?
		*"I'm 78 years old. These things have been left for the younger ages ago!"* (no answer)	Sexual	10. How would you describe the FREQUENCY of your erections during the last 4 weeks?
		*"Age must be considered"* (I never had an erection when I wanted one)	Sexual	10. How would you describe the FREQUENCY of your erections during the last 4 weeks?
	Spouse's illness (7)	*"Wife is in institutional care"* (no answers)	Sexual	8–10. Sexual function questions
		*"Wife's cancer surgery has caused this problem"* (moderate problem)	Sexual	12. Overall, how big a problem has your sexual function or lack of sexual function been for you during the last 4 weeks?
		*"Haven't had sexual activity for several years due to my wife's serious cardiovascular diseases. No masturbation either."* (no answers)	Sexual	8–11. All sexual function questions
Describing health conditions	Additional information about symptoms (73)	*"Usually when I take beer"* (more than once a week)	Urinary	1. Over the past 4 weeks, how often have you leaked urine?
		*"Incontinence only appears when coughing but not always (fierce coughing!)"* (total control)	Urinary	2. Which of the following best describes your urinary control during the last 4 weeks?
		*"Maybe not depression but thoughts tend to circle around cancer diagnosis"* (very small problem)	Hormonal	13 c. How big a problem during the last 4 weeks, if any, has feeling depressed been for you?
	Additional information about treatment (22)	*"Operation was done 1st of Aug 2018! Since then diaper consumption has increased"* (Chose two answer options: none and 3 or more pads a day)	Urinary	3. How many pads or adult diapers per day did you usually use to control leakage during the last 4 weeks?
		*"Hormone therapy since July 2018"* (very poor or none)	Sexual	8 a-b. How would you rate your ability to have an erection/reach orgasm during the last 4 weeks?
		*"I had surgery 1st of May which affects my answers"*	All domains	Comment written beside urinary incontinence and bowel symptom questions and again at the end of survey
Indicating possible sources of data validity issues	Erectile aid use (15)	*"Note! When I use erectile aid"* (I had an erection whenever I wanted one)	Sexual	10. How would you describe the FREQUENCY of your erections during the last 4 weeks?
		*"With the help of pills"* (firm enough for intercourse)	Sexual	9. How would you describe the usual QUALITY of your erections during the last 4 weeks?
		*"Good enough with erectile aid"* (not firm enough for any sexual activity)	Sexual	9. How would you describe the usual QUALITY of your erections during the last 4 weeks?
	Other illnesses (15)	*"Celiac disease"* (very small problem)	Bowel	7. Overall, how big a problem have your bowel habits been for you during the last 4 weeks?
		*"Furesis"* (very small problem)	Urinary	4 e. How big a problem, if any, has need to urinate frequently during the day been for you during the last 4 weeks?
		*"5 years ago a prolapsed disc, a spinal cord injury, paraplegia (surgery)"* (moderate problem)	Sexual	12. Overall, how big a problem has your sexual function or lack of sexual function been for you during the last 4 weeks?

### Analysis

We used inductive content analysis to examine participants’ comments regarding EPIC questions in our questionnaire. In content analysis, the content of a text is interpreted through a systematic classification process of coding and identifying patterns [see conventional content analysis in 11]. We followed Erlingsson and Brysiewicz’s [12] example of inductive content analysis, whereby a large amount of text is systematically transformed into a concise, organized summary of key results. Each step of the analysis increases the level of abstraction of the data, from literal content to latent meanings.

First we read through the primary data multiple times to gain a general understanding of how patients commented on our questionnaire. Each comment was divided into meaning units, coded according to content and the EPIC domain referred to (sexual, urinary, bowel, hormonal). Codes that were related to each other through their content were grouped into the same category [12]. Because no systematic studies of patients’ views on the EPIC questionnaire had previously been conducted, we did not use preconceived categories; instead we allowed categories to “flow from the data” [11]. Ten categories describing the content of participants’ comments were generated. These categories were further grouped into four themes demonstrating the patients’ purposes when commenting on EPIC questions (Table 2).

We also quantified the condensed meaning units we had generated to see which EPIC domain (sexual, urinary, bowel, hormonal) was most commented (Table 3). This quantitative approach to content analysis enabled us to explore the usage of certain comments [11], giving us information about which domains were probably the most difficult for respondents to answer. Content analysis was performed using ATLAS.ti version 8 (ATLAS.ti GmbH, Berlin, Germany) and IBM SPSS Statistics 27 (IBM Corp, Armonk, NY, USA) was used to describe study sample (Table 1).

**Table 3:** Number of comments referring to different EPIC domains

Theme	Category (number of comments)	Bowel	Urinary incontinence /irritative	Sexual	Hormonal
Indicating difficulties in answering	No attempts at erection (36)			36	
	Use of medical devices (16)	2	12	2	
	Reluctance to answer (10)	3	1	5	1
	Absence of a sexual relationship (6)			6	
Explaining life situations	Old age (9)			9	
	Spouse's illness (7)			7	
Describing health conditions	Additional information about symptoms (73)	12	31	18	12
	Additional information about treatment (22)	5	10	7	
Indicating possible sources of data validity issues	Erectile aid use (15)			15	
	Other illnesses (15)	10	1	1	3
	Total (209)	32	55	106	16

## Results

### Motives for commenting on EPIC questions

In the data, the patients commented frequently on the questions they had trouble answering. Several comments related to questions where the patient had chosen two or more answer options, or none. Many gave explanations for not answering a question. Also, patients felt it necessary to give a variety of descriptions of their symptoms and life circumstances, to convey a better understanding of their own situation. Additionally, participants described situations where their chosen answer might draw a false picture of prostate cancer treatment-related side effects. Sample extracts, and the categories and themes generated from the data, are shown in Table 2. Table 3 presents the division of different comment types for each EPIC domain.

#### Indicating difficulties in answering

In comments written on the form, participants described situations where they could not find a suitable response option. They either did not want to give any answer to the question, or there simply were none that fitted. A few men chose an answer option all the same, but their comment indicated that the choice was based on a guess. As seen in Table 3, participants had the most trouble answering sexuality-related items.

##### No attempts at erection

The second largest category in our analysis concerned the sexual domain (Table 3). There were two types of comments here: either the men stated that they had not had sexual activity, or they commented that they had not wanted an erection in the previous four weeks (Table 2). In either case the men could not know about their current erectile function, which led them to either skip the questions about sexual functioning or guess the answer.

##### Use of medical devices

Patients who had either an indwelling or a suprapubic urinary catheter (Cystofix®) could not find a suitable answer option to the urinary function questions. Some still reported having “total” urinary control, even if it was due to their medical device (Table 2). As shown in Table 3, having a urinary catheter also affects men’s answers to questions about sexual functioning: one participant commented that he could not function sexually due to his urinary catheter.

Patients who had a stoma connected to the bowel could not find an appropriate answer option to bowel function questions. In our data, only two patients reported having a colostomy. In cases of catheter or colostomy, questions related to urinary or bowel functioning were usually left unanswered.

##### Reluctance to answer

Some patients had clearly noted the question but decided not to answer. Three of these simply refused to answer a question, even though a suitable answer option was available. For example, in the first extract in Table 2, the patient wrote a comment describing his erectile function, but he did not choose any of the available answer options that would match the poor function described. Some men who declined to answer nonetheless addressed the question by writing an exclamation mark, a dash, or the word “NO!,” which we interpreted as a statement of not wanting to answer. Two of the three bowel function-related comments in this category clearly denoted that the participant did not know whether he had bloody stools (Table 3).

##### Absence of a sexual relationship

Some single men explained their unanswered sexual function questions by referring to their current relationship status. It appears that men with no sexual relationships found it especially difficult to find a suitable answer option, although the questionnaire itself only concerns objective erectile capability, not intercourse within a relationship. There was also one comment from a widower who had answered all the sexuality-related questions, but whose comment indicated that he had not attempted to have an erection recently (Table 2).

#### Explaining life situations

In EPIC-26’s sexual domain, some participants described life situations that affected the way they answered the sexuality-related questions. They gave reasons for their answers, and oftentimes explained why they had skipped a question.

##### Old age

Comments where patients mentioned their advanced age served two purposes. First, a few patients explained their poor erectile ability in terms of their high age. Second, high age was also used as an explanation for not wanting or trying to have an erection (Table 2). Four of the nine patients that wrote age-related comments did not answer the survey’s sexual function questions.

##### Spouse’s illness

Five men explained that they had skipped sexual function and/or bother questions because their spouses had illnesses that prevented them from being sexually active in their relationship (Table 2). Similarly, in the category “absence of a sexual relationship,” the men that wrote such comments had not tried to get an erection because there was no partner capable of sex. Both categories highlight that these men perceive sex as part of a relationship rather than in terms of individual sexuality, and that sexual activity means primarily intercourse.

Sexual bother questions evoked two comments where the patient explained his level of sexual bother in terms of his spouse’s health situation. In both cases, the spouse herself had cancer, which affected the man’s sexuality. With these comments, the two men indicated that their level of sexual bother was due to something other than prostate cancer treatment.

#### Describing health conditions

The theme that included the most comments and condensed meaning units pertained to the patients’ plentiful additional information about their symptoms or treatment (Table 3). Oftentimes, with these comments participants indicated changes in symptoms over time, from treatment or from the nature of their daily activities. The EPIC-26 urinary domain clearly produced the most additional information (Table 3).

##### Additional information about symptoms

Typically, patients’ comments provided more detailed information about the quality and severity of their symptoms, as well as about what provoked the symptom (usually urinary incontinence). A few participants also mentioned other symptoms that were not asked about in the survey but which they considered important. Some comments also described a change in symptoms during past weeks or months. This category was the only one to produce a notable number of comments in EPIC-26’s hormonal domain, which otherwise was rarely commented on (Table 3).

##### Additional information about treatment

Information about treatment was usually added when the participant wished to denote a change in the occurrence or severity of symptoms after treatment. As seen in Table 2, sometimes the patient chose more than one answer option to indicate such changes. For instance, patients used treatment information to explain their poor erectile ability or their level of incontinence. Many mentioned the dates when their prostate cancer treatment was received, even though we had informed them that we would collect treatment-related information from the hospital register. Only three participants added information about medication for bowel or urinary symptoms, describing the medication’s effect on their symptoms.

#### Indicating possible sources of data validity issues

Patients also gave information about other illnesses or the use of erectile aids, which they thought might lead to wrong assumptions about the causes of certain answers.

##### Erectile aid use

Especially in the case of erectile aid use, men in similar situations could end up giving very different answers to the same erectile function questions, depending on whether they answered these questions with or without considering the effect of erectile dysfunction medication (Table 2). One patient directly stated his opinion that erectile aid use should be considered in the survey.

##### Other illnesses

Men gave information about symptoms that were not related to their prostate cancer or its treatment but were nevertheless covered by the survey. Four of the 10 comments regarding the bowel domain (Table 3) in this category related to hemorrhoids that caused bloody stools. Celiac disease and cancer of the rectum caused similar bowel symptoms to radiation therapy. Three patients reported on medication that caused either urinary or bowel symptoms (e.g., diuretic medication, Table 2). As shown in Table 3, the survey’s bowel domain was the most affected by other illnesses that influenced how participants answered the questionnaire.

### Comments provoked by different EPIC domains

As reported above, the sexual domain provoked the most comments of the four domains: over 50% of all comments related to the sexual domain (Table 3). Within the sexual domain, men’s main reasons for writing comments in the margin were (1) they had not attempted to get an erection, and therefore could not know about the quality or frequency of their erections; (2) they wished to share additional information about the severity of their erectile dysfunction, and any changes to that function due to their treatment or illness; (3) they wanted to inform us whether they had taken their use of erectile aid into account when answering sexual function questions (Table 3).

The second most comment-provoking domain was the urinary domain, including questions related to both urinary incontinence and urinary irritative symptoms and bother. More than a quarter of all comments were in this domain (Table 3). The main causes of comments on urinary function questions were (1) the need to give additional information about the severity and quality of urinary symptoms; (2) the need to indicate that there was no suitable answer option for someone with a urinary catheter; (3) the need to describe a change in urinary function due to treatment. Of the 73 meaning units in the large category “additional information about symptoms,” 42% related to the urinary domain (Table 3).

The bowel domain provoked 15% of all the comments analyzed in our study. The most important reasons for comments on questions about bowel function and bother were (1) the need to give additional information about symptoms; (2) the need to indicate that the prevalent symptom was caused by an illness other than prostate cancer (Table 3).

The hormonal domain produced only 8% of all comments (Table 3). As with other domains, the hormonal comments encompassed some patients’ need to further describe their symptoms.

## Discussion

This study aimed to depict patients’ perspectives on the use of EPIC-26 to measure HRQoL after prostate cancer diagnosis. In respect to further enhancing the reliability and validity of this widely used instrument, we were interested in respondents’ experiences of the survey. Here, we discuss our key observations and provide suggestions for future directions in the development of EPIC-26.

Brundage et al. [10] recognized the need to determine strategies to minimize missing data in EPIC-CP, and wished for information regarding why patients skip questions. They also noted that missing data were especially a problem in the survey’s sexual domain. Similarly, our participants gave several reasons for skipping questions, and the sexual domain in particular produced more than half of all comments. A major reason for skipping items was that the question was not relevant to the patient if he had had no sexual activity during the previous four weeks. The problems with answering erectile function questions lie in the questions’ presumptions. Question 10 assumes that the respondent wants to have an erection (see Appendix), but this is not always the case. It is also important to acknowledge that if one has not been sexually active in the previous four weeks, it is difficult to estimate one’s erectile ability (questions 8–9). Our study supports previous findings that patients require a “not applicable” category for erectile function questions [9]. The same issue does not pertain to the sexual bother question, since it does not presuppose sexual activity or desire.

Erectile medication use is not considered in this disease-specific instrument. In addition to the difficulty it causes patients, who have to decide whether or not they are expected to take their medicine use into account, this shortcoming affects the interpretation and reliability of the statistical data received. First, any difficulty in completing a survey increases the risk of missing data. Second, if the patient does not recall whether he took his erectile aid use into account during earlier survey rounds, he may use different strategies to answer the same question in different rounds; this will weaken the test–retest reliability, although EPIC-26’s reliability has been confirmed as sufficient [7]. Hence, the use of erectile aid should be incorporated into this instrument targeted at prostate cancer patients.

In the urinary domain, a clear reason for skipping questions was the use of an indwelling or suprapubic urinary catheter, which made the questions irrelevant to the patient. Due to prostate cancer treatment or progression of the cancer, catheter use is often required. Hence, it would be reasonable to note catheter use in the questionnaire. Although the same issue emerged with colostomies, the number of these patients is smaller, and it might not be so reasonable to take this matter into account and thereby increase the length of the survey.

Because quantitative surveys cannot consider all the possible factors that patients might consider important [2], there are always going to be comments written in the margins of pen-and-paper surveys. Patients’ descriptions of their health condition generated the largest theme in our study, illustrating patients’ natural need to explain and clarify their life experiences. Compared with electronic surveys with no space for additional information, the ability to write in a paper margin might encourage respondents to answer a survey, since there is space for explanation with every question. That said, a computerized version of EPIC-26 has also proved to be highly accepted by patients in clinical practice [13]. As we consider these e-versions useful tools, free-text fields are important in online surveys where there is otherwise no natural space for comments. Allowing comments also allows transpiring of previously unknown problems within the survey and participants development suggestions that are valuable for the development of the measure.

## Conclusion

This study sheds light on the challenges related to measuring HRQoL with a survey that focuses on disease-related symptoms and bother. Our study concerns mainly physical dimension of patients’ overall QoL with the main findings directing to sexual and urinary functioning. Our findings regarding EPIC-26 are in line with the previous results on EPIC-CP, in that patients are mainly willing to report their symptoms [10]. The challenges related to answering EPIC-26 relate to a lack of suitable answer options, which might weaken this instrument’s content validity if the items are insufficiently comprehensive or relevant for certain patients. Our conclusion is that considering the use of erectile medication and adding a new category to sexual function questions 8–10 would decrease the skipping of sexuality-related questions and further improve the quality of EPIC-26. Similarly, as mentioned earlier, a consideration of the use of urinary catheters would probably reduce missing data on items 1–4. Certainly, further studies are required to examine the statistical aspects of these suggested developments. Also, broader statistical analyses on sociodemographic or clinical factors related to skipping questions in EPIC would be important to gain more knowledge about which patient groups struggle the most in finding suitable answer options.

## Supplementary Information

Below is the link to the electronic supplementary material.

Supplementary file1 (PDF 93 kb)

## Data Availability

The datasets analyzed during the current study are not publicly available due to restrictions in research permission but are available from the corresponding author on reasonable request.
