# Supplementary material for: The expanded prostate cancer index composite short form (EPIC-26) for measuring health-related quality of life: content analysis of patients’ spontaneous comments written in survey margins
Source: Qual Life Res. 2021 Jul 21;31(3):855–64. doi: 10.1007/s11136-021-02940-z (PMC8921047; doi:10.1007/s11136-021-02940-z)
Supplement: Supplementary file 1 — Supplementary file1 (PDF 93 kb) [file 11136_2021_2940_MOESM1_ESM.pdf]

EPIC-26

The Expanded Prostate Cancer Index Composite

Short Form

This questionnaire is designed to measure Quality of Life issues in patients with Prostate cancer. To help us get the most accurate measurement, it is important that you answer all questions honestly and completely.

Remember, as with all medical records, information contained within this survey will remain strictly confidential.

Today's Date (please enter date when survey completed): Month________Day________Year________

Name (optional): ____

Date of Birth (optional): Month__________Day___________Year__________

Do Not Mark in This Space

1. Over the **past 4 weeks**, how often have you leaked urine?

More than once a day...................... 1
About once a day............................ 2
More than once a week.................... 3 (Circle one number) 23/
About once a week...........................4
Rarely or never............................... 5

2. Which of the following best describes your urinary control **during the last 4 weeks**?

No urinary control whatsoever........... 1
Frequent dribbling.. 2 (Circle one number) 26/
Occasional dribbling.. 3
Total control.. 4

3. How many pads or adult diapers per day did you usually use to control leakage **during the last 4 weeks**?

None 0
1 pad per day.. 1
2 pads per day.. 2 (Circle one number) 27/
3 or more pads per day... 3

4. How big a problem, if any, has each of the following been for you **during the last 4 weeks**?

(Circle one number on each line)

	No Problem	Very Small Problem	Small Problem	Moderate Problem	Big Problem	
a. Dripping or leaking urine	0	1	2	3	4	28/
b. Pain or burning on urination.....	0	1	2	3	4	29/
c. Bleeding with urination.............	0	1	2	3	4	30/
d. Weak urine stream or incomplete emptying	0	1	2	3	4	31/
e. Need to urinate frequently during the day....................................	0	1	2	3	4	33/

5. Overall, how big a problem has your urinary function been for you **during the last 4 weeks**?

No problem..................................... 1
Very small problem.......................... 2
Small problem..................................3 (Circle one number) 34/
Moderate problem........................... 4
Big problem.................................... 5

Copyright 2002. The University of Michigan. All rights reserved.

Do Not Mark in This Space

6. How big a problem, if any, has each of the following been for you? (Circle one number on each line)

	No Problem	Very Small Problem	Small Problem	Moderate Problem	Big Problem	
a. Urgency to have a bowel movement	0	1	2	3	4	49/
b. Increased frequency of bowel movements....................	0	1	2	3	4	50/
c. Losing control of your stools......	0	1	2	3	4	52/
d. Bloody stools	0	1	2	3	4	53/
e. Abdominal/ Pelvic/Rectal pain...	0	1	2	3	4	54/

7. Overall, how big a problem have your bowel habits been for you **during the last 4 weeks**?

No problem.................................... 1
Very small problem......................... 2
Small problem.................................3 (Circle one number) 55/
Moderate problem.......................... 4
Big problem.................................... 5

8. How would you rate each of the following **during the last 4 weeks**? (Circle one number on each line)

	Very Poor to None	Poor	Fair	Good	Very Good	
a. Your ability to have an erection?...............................	1	2	3	4	5	57/
b. Your ability to reach orgasm (climax)?......................	1	2	3	4	5	58/

9. How would you describe the usual QUALITY of your erections **during the last 4 weeks**?

None at all.. 1
Not firm enough for any sexual activity... 2
Firm enough for masturbation and foreplay only.............................. 3 (Circle one number) 59/
Firm enough for intercourse.. 4

10. How would you describe the FREQUENCY of your erections **during the last 4 weeks**?

I NEVER had an erection when I wanted one.................................. 1
I had an erection LESS THAN HALF the time I wanted one............ 2
I had an erection ABOUT HALF the time I wanted one .... 3 (Circle one number) 60/
I had an erection MORE THAN HALF the time I wanted one........... 4
I had an erection WHENEVER I wanted one.................................... 5

Copyright 2002. The University of Michigan. All rights reserved.

Do Not Mark in This Space

11. Overall, how would you rate your ability to function sexually **during the last 4 weeks**?

Very poor	1		
Poor	2		
Fair	3	(Circle one number)	64/
Good	4		
Very good	5		

12. Overall, how big a problem has your sexual function or lack of sexual function been for you **during the last 4 weeks**?

No problem	1		
Very small problem	2		
Small problem	3	(Circle one number)	68/
Moderate problem	4		
Big problem	5		

13. How big a problem **during the last 4 weeks**, if any, has each of the following been for you? (Circle one number on each line)

	No Problem	Very Small Problem	Small Problem	Moderate Problem	Big Problem	
a. Hot flashes	0	1	2	3	4	74/
b. Breast tenderness/enlargement	0	1	2	3	4	75/
c. Feeling depressed	0	1	2	3	4	77/
d. Lack of energy	0	1	2	3	4	78/
e. Change in body weight	0	1	2	3	4	79/

THANK YOU VERY MUCH!!

Copyright 2002. The University of Michigan. All rights reserved.
